# Performance of Two Southern California Benthic Community Condition Indices Using Species Abundance and Presence-Only Data: Relevance to DNA Barcoding

**DOI:** 10.1371/journal.pone.0040875

**Published:** 2012-08-07

**Authors:** J. Ananda Ranasinghe, Eric D. Stein, Peter E. Miller, Stephen B. Weisberg

**Affiliations:** 1 Southern California Coastal Water Research Project, Costa Mesa, California, United States of America; 2 Canadian Centre for DNA Barcoding, Biodiversity Institute of Ontario, University of Guelph, Guelph, Canada; Brigham Young University, United States of America

## Abstract

DNA barcoding, as it is currently employed, enhances use of marine benthic macrofauna as environmental condition indicators by improving the speed and accuracy of the underlying taxonomic identifications. The next generation of barcoding applications, processing bulk environmental samples, will likely only provide presence information. However, macrofauna indices presently used to interpret these data are based on species abundances. To assess the importance of this difference, we evaluated the performance of the Southern California Benthic Response Index (BRI) and the AZTI Marine Biotic Index (AMBI) when species abundance data were removed from their calculation. Presence only versions of these two indices were created by eliminating abundance weighting while preserving species identity. Associations between the presence and abundance BRI, and the presence and abundance AMBI were highly significant, with correlation coefficients of 0.99 and 0.81, respectively. The presence versions validated almost equally to the abundance-based indices when applied to the spatial and the temporal monitoring data used to validate the original indices. Simulations in which taxa were systematically removed from calculation of the indices were also conducted to assess how large the barcode library must be for the indices to be effective. Correlation between the BRI-P and BRI remained above 0.9 with only 370 species in the library and reducing the number of species to 450 had almost no effect on correlation between the presence and abundance versions of the AMBI.

## Introduction

Marine benthic macrofauna are frequently used as indicators of environmental condition because they reside in sediments where contaminants accumulate and their immobility allows them to integrate exposure at a site [1]–[3]. Benthic community composition is typically summarized using benthic indices that allow easy communication of complex biological information as a single number that ranks sites on a scale from good to bad. These index values allow managers to prioritize impacted sites, track trends over time, or correlate biological responses with stressor data.

**Figure 1:**
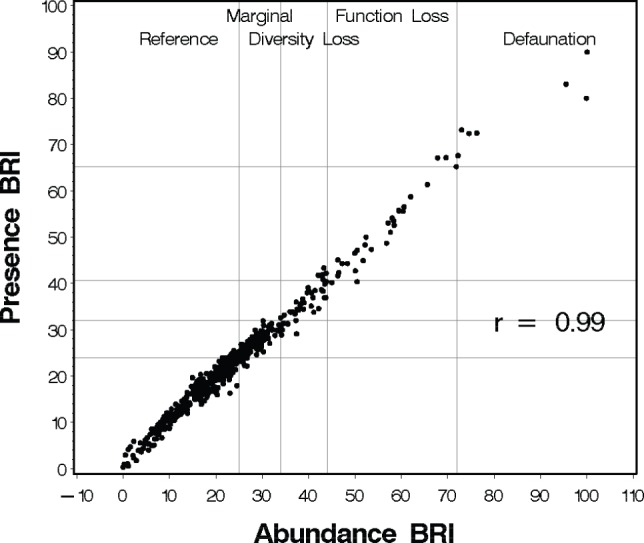
Relationship between Presence BRI (BRI-P) and the original abundance BRI. Vertical and horizontal lines indicate assessment thresholds for the BRI [12] and BRI-P (Table 1), respectively.

**Table 1:** Presence BRI assessment categories, assessment thresholds, and percentage of samples in agreement with abundance BRI assessment category in the 493 sample calibration data.

Assessment Category	BRI-P	Samples	Samples in same BRI category	
			n	%
Reference	≤24	304	298	98.0
Marginal	>24 to ≤32	114	92	80.7
Diversity Loss	>32 to ≤40.7	34	31	91.2
Community Function Loss	>40.7 to ≤65.3	32	25	78.1
Defaunation	≥ 65.3	9	7	77.8
Total		493	453	91.9

Challenges in using benthos as indicators are the cost, time and error associated with identifying the biota. Organisms must be manually separated from the sediment, which can take more than a day and is subject to underestimation as some remain hidden among the debris [4]. Every captured organism must then be identified, typically to species, which requires highly skilled taxonomists that provide expertise over the range of different taxonomic groups. This adds substantial cost and is subject to error, particularly when the specimens are damaged or immature life stages are present.

**Figure 2:**
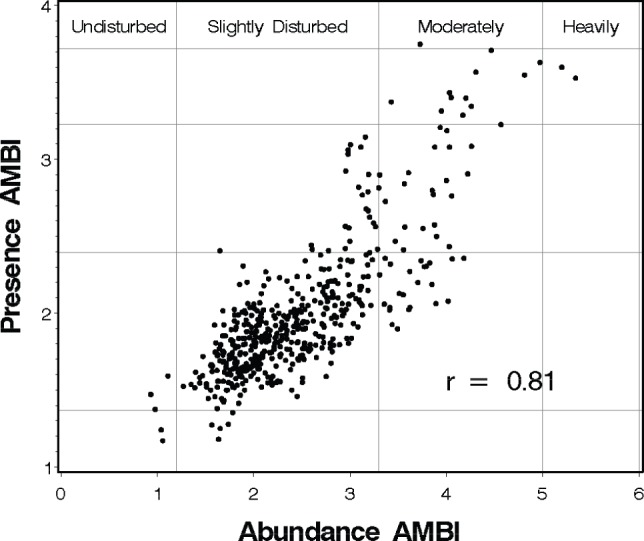
Relationship between Presence AMBI (AMBI-P) and the original abundance AMBI. Vertical and horizontal lines indicate assessment thresholds for the AMBI [13] and AMBI-P (Table 4), respectively.

**Table 2:** Presence AMBI assessment categories, assessment thresholds, and percentage of samples in agreement with abundance AMBI assessment category in the 493 sample calibration data.

Assessment Category	AMBI-P	Samples	Samples in same AMBI category	
			n	%
Undisturbed	≤1.37	7	2	28.6
Slightly Disturbed	>1.37 to ≤2.40	425	399	93.9
Moderately Disturbed	>2.40 to ≤3.23	47	23	48.9
Heavily Disturbed	>3.23 to ≤3.72	13	2	15.3
Extremely Disturbed	>3.72	1	0	0.0
Total		493	426	86.4

DNA barcoding has the potential to increase the speed, accuracy and resolution of species identification [5]–[7]. Barcoding involves identifying species based on a short gene sequence from a standardized portion in the genome, and for animals this is the mitochondrial cytochrome c oxidase 1 gene (CO1). Using standard molecular biology tools, DNA is extracted from the specimen tissue, and a 658 base pair region of the CO1 gene is amplified by polymerase chain reaction and sequenced [8]. DNA from unknown specimens collected in benthic samples can be identified by comparing their barcode sequences to the Barcode of Life Data Systems (BOLD, http://www.boldsystems.org) reference library.

**Figure 3:**
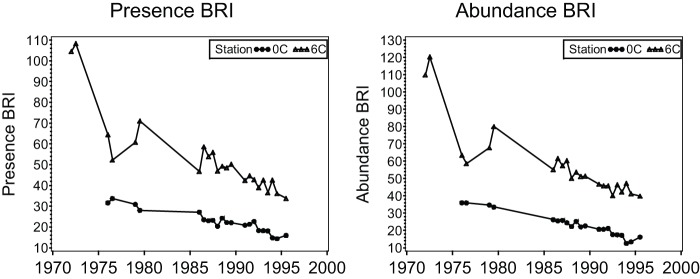
Application of the Presence BRI and BRI to outfall monitoring data from 1972 to 1995 at Los Angeles County Station 6C (2.2 km from the outfall) and Station 0C (14.7 km from the outfall).

**Figure 4:**
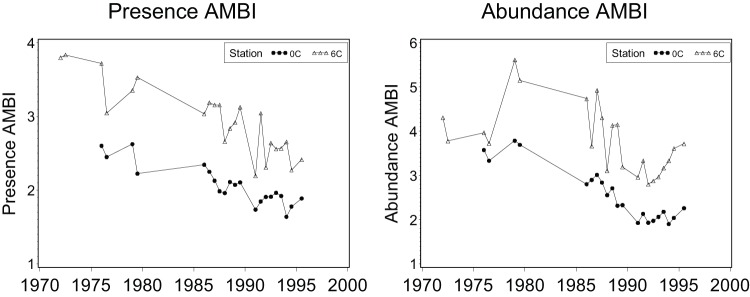
Application of the Presence AMBI and AMBI to outfall monitoring data from 1972 to 1995 at Los Angeles County Station 6C (2.2 km from the outfall) and Station 0C (14.7 km from the outfall).

Traditional DNA barcoding is based on Sanger sequencing in which each specimen is processed individually. Next-generation sequencing has the potential to further reduce the time and cost for processing through bulk processing [9], [10], in which the entire sample is homogenized, tissue lysed, DNA extracted and the species composition of the entire sample determined using metagenetic analysis of one or more markers. While this promises greater speed and lower costs [11], it produces presence-only information and prevailing benthic condition indices require abundance for each species. Unlike analysis of prokaryotes where number of reads can be used as a surrogate for number of phylotypes, the same assumption cannot be made for next-generation sequencing of multicellular organisms. Another consideration is that barcoding provides identification of unknown specimens by matching them to species in the reference library. Therefore, the reference library must contain a sufficient number of species to allow environmental samples to be analyzed to a level where there is enough taxonomic resolution to apply commonly used benthic indices. Here we redevelop presence-only versions of two benthic condition indices used for assessments in southern California and compare their performance to their abundance-based counterparts. Second, we evaluate the minimum number of taxa needed in a reference library to calculate a credible presence-based index.

**Figure 5:**
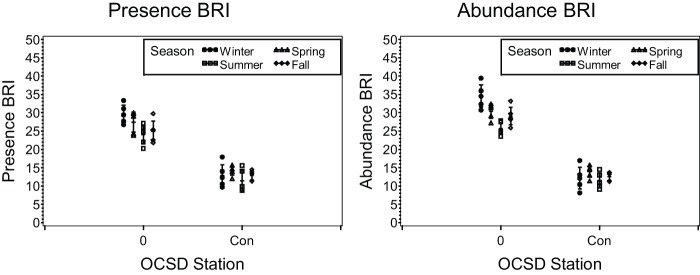
Application of the Presence BRI and BRI to 1990 seasonal outfall monitoring data at Orange County Station 0 (adjacent to the outfall) and Station Con (8 km from the outfall).

**Figure 6:**
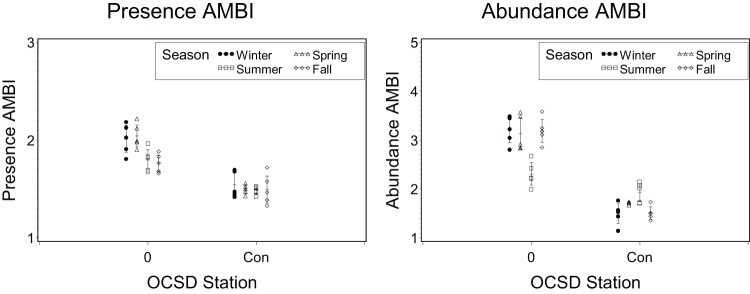
Application of the Presence AMBI and AMBI to 1990 seasonal outfall monitoring data at Orange County Station 0 (adjacent to the outfall) and Station Con (8 km from the outfall).

## Methods

The two indices used to compare performance between abundance and presence-only derivations were the Benthic Response Index (BRI) [12] and the AZTI Marine Biotic Index (AMBI) [13]. The BRI is based on the abundance-weighted average pollution tolerance of species in a sample. The pollution tolerance scores (“p-values”) are developed using ordination analysis to place sites along a pollution disturbance gradient, with pollution tolerance scores assigned to each species based on the position of its peak abundance along the gradient. Lower scores indicate sensitive species and higher scores indicate pollution tolerant species. The AMBI is also based on abundance-weighted pollution tolerances of species that are present, but tolerance is expressed categorically as one of five ecological groups. Species are assigned to ecological groups based on consensus expert judgment and assignments are transferrable among geographies.

**Figure 7:**
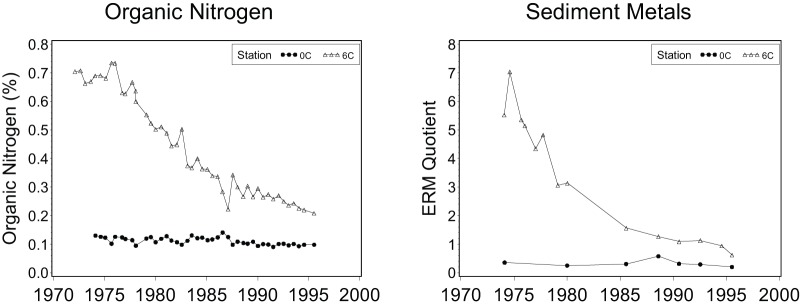
Sediment metal and organic nitrogen concentrations from 1972 to 1995 at Los Angeles County Stations 6C and 0C, for which benthic index data are presented in **Figures 3** and **4**. For each sample, concentrations of eight metals are integrated as mean effects range median (ERM, [17]) quotients. For each metal, the ERM quotient measures the ratio of the observed concentration to the value at which biological effects are likely, and the mean ERM quotient for the eight metals is an integrated measure of the likelihood of contaminant effects.

**Table 3:** Pearson correlation coefficients (r) between benthic indices and measures of pollution at Los Angeles County Stations 6C and 0C from 1972 to 1995.

Benthic Index	Organic Nitrogen			Mean ERM quotient			
	r	p	n		r	p	n
BRI-P	0.91	<0.0001	23		0.82	<0.01	9
BRI	0.90	<0.0001	23		0.78	<0.05	9
AMBI-P	0.91	<0.0001	23		0.89	<0.01	9
AMBI	0.69	<0.001	23		0.63	0.07	9

Organic nitrogen is a measure of eutrophication and the mean ERM [17] quotient is a measure of sediment contamination. For each of eight measured metals, the ERM quotient measures the ratio of the observed concentration to the value at which biological effects are likely, and the mean ERM quotient for the eight metals is an integrated measure of the likelihood of contaminant effects.

The presence BRI (BRI-P) was calculated using the tolerance scores of Smith *et al.* [12], but the average tolerance score for the BRI-P was calculated as the sum of species tolerance scores divided by the number of taxa with tolerance scores, without abundance weighting. Assessment thresholds for the resulting index were then developed by regression against the BRI and selecting thresholds corresponding to the original BRI assessment categories: (i) natural benthic assemblages; (ii) the loss of biodiversity, above which 25% of the species pool occurring in reference samples no longer occurred; (iii) loss of community function, where echinoderms and arthropods were lost from the assemblage; and (iv) defaunation, where 90% of the species pool in reference samples no longer occurred.

**Table 4:** Effect of reductions in numbers of taxa on the Benthic Response Index (BRI).

Percentage of species with tolerance scores included in BRI-P	No of species with tolerance scores included in BRI-P	BRI-P vs. BRI Pearson Correlation Coefficient	No. of stations with BRI-P values
10	46	0.67	380
20	92	0.76	457
30	139	0.80	488
40	185	0.83	490
50	231	0.89	493
60	277	0.87	493
70	323	0.88	492
80	370	0.90	493
90	416	0.92	493
100	462	0.99	493

BRI-P values could not be calculated for stations where no species with tolerance scores were present in the data after the reduction.

**Table 5:** Effect of reductions in numbers of taxa on the AZTI Marine Biotic Index (AMBI).

Percentage of species with ecological group assignments included in theAMBI-P	No of species with ecological group assignments included in the AMBI-P	AMBI-P vs. AMBI Pearson Correlation Coefficient	No. of stations with AMBI-P values
10	65	0.49	493
20	129	0.65	493
30	193	0.66	493
40	257	0.75	493
50	321	0.76	493
60	385	0.76	493
70	449	0.80	492
80	513	0.81	493
90	577	0.81	493
100	641	0.81	493

The presence AMBI (AMBI-P) calculations followed Borja *et al.* [13], with the ecological group scores expressed as the percentage of species in each ecological group without abundance weighting. Assessment thresholds for the AMBI-P were developed by regression against the AMBI and selecting thresholds corresponding to the original AMBI assessment categories: (i) undisturbed, (ii) slightly disturbed, (iii) moderately disturbed, (iv) heavily disturbed, and (v) extremely disturbed.

**Figure 8:**
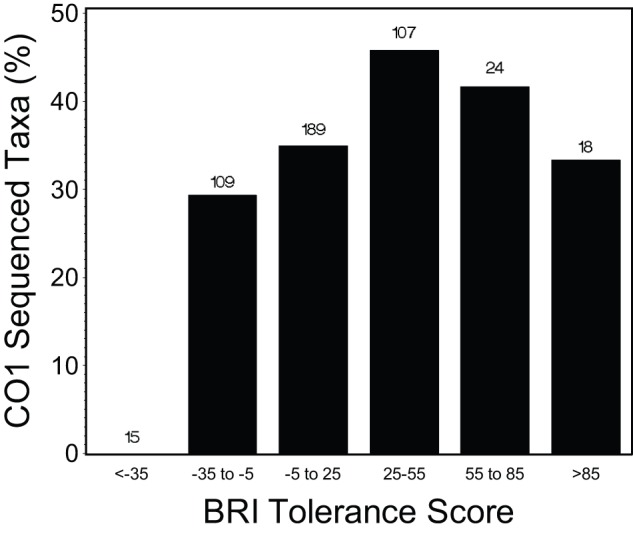
Percentages of taxa with BRI tolerance scores which have CO1 sequences available in the Barcode Of Life Database. Taxa were segregated into six categories along the disturbance gradient by BRI tolerance scores. The total number of taxa with tolerance scores in each category are presented above the bar indicating sequence availability. Barcodes were available for 163 of 462 taxa with tolerance scores.

**Table 6:** Barcoding success rates.

Taxon	No. of species attempted for barcoding	Species with sequences >500 base pairs (%)
Cnidaria	4	50.0
Platyhelminthes, Turbellaria	1	0.0
Nemertea, Anopla	4	50.0
Sipuncula	2	50.0
Echiura	1	0.0
Mollusca, Gastropoda	15	40.0
Mollusca, Bivalvia	17	41.1
Mollusca, Scaphopoda	2	100.0
Annelida, Polychaeta	126	62.7
Arthropoda, Pycnogonida	1	100.0
Arthropoda, Ostracoda	3	100.0
Arthropoda, Malacostraca	45	71.1
Brachiopoda	1	100.0
Echinodermata	2	50.0
Hemichordata	1	0.0
**Total**	**225**	**60.9**

The percentages of species yielding COI sequences of more than 500 base pairs are presented.

### Assessing performance of the Presence BRI and the Presence AMBI

Performance of the BRI-P and AMBI-P were assessed using the two southern California data sets used to validate the original BRI, both of which were independent of the calibration data used to develop the indices. The first tested whether the BRI-P and AMBI-P reproduced known temporal gradients of benthic conditions over several years near a southern California waste-water outfall using data from two Los Angeles County Sanitation Districts monitoring sites which were sampled monthly since 1972. The first site, Station 6C (located 2220 m from the outfall) was severely impacted in the early 1970 s and has improved since that time [14], [15]. The second site, Station 0C (located 14,720 m from the outfall) was less affected than Station 6C, but has also improved. The hypothesis was that BRI-P index values should decrease over time at Stations 6C and 0C and that index values will be higher and decrease more at Station 6C than at Station 0C.

The second data set was used to test whether the presence indices reproduced a known spatial gradient between two stations on the 60 m isobath from the Orange County Sanitation Districts outfall. Previous studies have shown that Station 0 located near the outfall has altered species composition in comparison to reference Station Con, which is located 7840 m from the outfall [16].

The first data set was also used to confirm that the observed benthic index patterns were likely responses to pollution. Sediment contaminant effects were tested by comparing benthic index values to the combined effects of eight metals (arsenic, cadmium, chromium, copper, lead, mercury, silver, and zinc), and eutrophication effects were tested using organic nitrogen concentrations. The effects of the eight metals were combined by calculating mean effects range median (ERM [17]) quotients. For each metal, the ERM quotient measures the ratio of the observed concentration to the value at which biological effects are likely, and the mean ERM quotient for the eight metals is an integrated measure of the likelihood of contaminant effects. Pearson correlation coefficients were used to measure associations between annual means for the BRI-P and the AMBI-P benthic indices and mean ERM quotients and organic nitrogen concentrations. The hypothesis was that the benthic indices and one or both pollution measures would be significantly correlated.

### Effect of Reducing the Number of Taxa

To assess how large the barcode library must be for an effective index, changes in Pearson correlation coefficients between the BRI-P and the BRI and the AMBI-P and AMBI were determined as taxa were systematically removed from calculation of the indices. To accomplish this, taxa were ranked according to their tolerance scores (BRI) or ecological groups and abundance (AMBI) and selected for removal at even intervals. This process was repeated with increasing percentages of taxa removed from the calculation.

## Results

There was a strong linear relationship between the BRI-P and BRI (Figure 1) with a Pearson correlation coefficient of 0.99 (p<0.0001). Application of the BRI-P assessment thresholds calculated by applying the equation from the calibration linear regression analysis (Table 1) resulted in 92% of the 493 calibration samples assigned to the same assessment category by both indices. Where assessment categories disagreed, the samples were always in adjacent categories, with no samples disagreeing by more than one category. The high agreement is reflected in the strong linear relationship in Figure 1.

The relationship between the AMBI-P and AMBI (Figure 2) was also significant, with a Pearson correlation coefficient of 0.81 (p<0.0001). Application of AMBI-P assessment thresholds (Table 2) resulted in 86.4% of the calibration samples assigned to the same assessment category by both indices.

Application of the BRI-P to the validation data resulted in patterns almost identical to the original abundance BRI results for both validation data sets, and AMBI-P patterns were similar to AMBI results. For the Los Angeles County outfall temporal data, the BRI-P (Figure 3) and the AMBI-P (Figure 4) accurately reflected the severe impacts at Los Angeles County Station 6C in the early 1970's, as well as the substantial improvement that occurred over time. Both the BRI-P and AMBI-P also correctly identified Station 0C, located 14.7 km from the outfall, as less affected than Station 6C with only slight biodiversity loss in the early 1970's and improvement to reference conditions in the 1990's.

The BRI-P (Figure 5) and AMBI-P (Figure 6) also correctly identified the spatial patterns near the Orange County outfall, with Station 0 located adjacent to the outfall having poorer benthic condition relative to Station Con, which is located 7.8 km away. Both indices also retained similar among-station differences across all seasons, as was observed in the original BRI validation.

The BRI-P and AMBI-P index values for the Los Angeles County outfall temporal data were significantly correlated with mean ERM quotients and sediment organic nitrogen concentrations (Table 3), indicating that index values were likely responding to anthropogenic pollution. Correlation coefficients for the presence based indices were comparable or stronger than the abundance based formulations. The time series of sediment metal and organic nitrogen concentration measurements at the two stations also resulted in patterns similar to the BRI-P and the AMBI-P time series (Figure 7).

Reducing the number of species included in BRI-P calculations resulted in a correlation greater than 0.9 relative to the abundance BRI even when 20% of the species with tolerance scores were removed (Table 4). For the AMBI-P, removing up to 30% of the species with AMBI ecological group assignments had negligible effects on the correlation between the AMBI-P and AMBI (Table 5).

## Discussion

The benthic community gradients used to validate the original BRI were reproduced closely by the BRI-P and AMBI-P. BRI-P and AMBI-P values at the two temporal validation stations were also highly correlated with sediment metal and organic nitrogen concentrations and patterns over time closely matched, indicating that the benthic indices were likely responding to anthropogenic pollution effects. There were only minor reductions in classification efficiency using presence-only information and no changes in the patterns or magnitudes of difference among sites. Reductions in index performance were small relative to previous attempts to reduce processing cost by identifying taxa only to genus or family level [18]–[20]. DNA barcoding provides the potential for improving index resolution by identifying cryptic species and clarifying species complexes that are inseparable by traditional morphological taxonomy methods [21], [22].

Similarity in performance between the BRI-P, AMBI-P and the traditional BRI results because the indices rely on composition of the whole community, not just the dominant taxa. Smith *et al.* [12] found that even removing the top ten most abundant taxa had minimal effect on the performance of the abundance BRI. Reliance on the entire community is enhanced in the BRI by use of a cube-root transformation which lessens the influence of abundant species, selected by Smith *et al.* [12] using an optimization algorithm to maximize index performance. This is similar to the findings of Warwick *et al.* [23] and Teixeira *et al.* [24] that performance of the AMBI is enhanced by transformations that reduce the influence of dominant taxa.

While the presence based approach worked well for the BRI and AMBI, it is unclear whether it will be equally successful in all situations. For instance, this study was conducted in euhaline water where there was an average of 67 taxa per sample; the presence-only index may be less sensitive in oligohaline waters where index development is more challenging because there are typically fewer than ten taxa per sample, even at reference sites [25], [26]. It might also not work as well in locations with more severe benthic community effects, such as in Chesapeake Bay where hypoxia leads to substantial reductions in benthic abundance. The disturbance gradient effects in this study were mostly limited to replacement of pollution sensitive with pollution tolerant taxa and indices that rely more heavily on dominance and diversity measures, which require abundance information, may be necessary to capture the larger range of effects. Still, species sensitivity and tolerance to disturbance are more robust measures of benthic community condition than abundance, diversity, or other community measures in multimetric indices [27]–[29].

Development of a locally relevant DNA reference library has been suggested as a potential impediment to incorporation of molecular methods in routine bioassessment, but our results suggest this should not be problematic. There are DNA barcode sequences for 159 southern California taxa with index tolerance scores already cataloged in BOLD and we found that reliable indices can be produced with a reference barcode library of less than 400 taxa. Moreover, the 159 taxa are relatively evenly distributed across the disturbance gradient (Figure 8). Of 225 species for which DNA barcoding was attempted, before February 2012, barcoding was successful for 61.8%, including 73.5%, 64.3%, and 44.1%, of arthropod, polychaete and mollusc species, respectively (Table 6). Many recent barcoding failures are due to molecular and biochemistry challenges that are in the process of being solved (for example, by improving primer sets). Success rates are high enough that we don't expect barcoding failure to be a major impediment and they don't affect our conclusions about performance of presence-based metrics.

There are several aspects of molecular methods that need to be investigated before they can be adopted for biological assessments, such as improved understanding of intraspecific genetic variation within groups currently considered as single species. There are also issues associated with sample handling. For instance, formalin, the presently used preservative for benthic studies, does not preserve DNA and the typical methods for preserving DNA are not conducive to field collections. However, the lack of quantification in species identification does not seem to be an impediment to adoption of molecular methods in biological assessments.
